# Metatranscriptomic analysis uncovers prevalent viral ORFs compatible with mitochondrial translation

**DOI:** 10.1128/msystems.01002-22

**Published:** 2023-05-18

**Authors:** Adam Begeman, Artem Babaian, Samantha C. Lewis

**Affiliations:** 1 Department of Molecular and Cell Biology, University of California, Berkeley, California, USA; 2 Department of Molecular Genetics, University of Toronto, Toronto, Ontario, Canada; 3 Donnelly Centre for Cellular and Biomolecular Research, University of Toronto, Toronto, Ontario, Canada; University of Wisconsin-Madison, Madison, Wisconsin, USA

**Keywords:** translation, metagenomics, RNA virus, mitochondria, mitovirus, virus evolution

## Abstract

RNA viruses are ubiquitous components of the global virosphere, yet relatively little is known about their genetic diversity or the cellular mechanisms by which they exploit the biology of their diverse eukaryotic hosts. A hallmark of (+)ssRNA (positive single-stranded RNA) viruses is the ability to remodel host endomembranes for their own replication. However, the subcellular interplay between RNA viruses and host organelles that harbor gene expression systems, such as mitochondria, is complex and poorly understood. Here we report the discovery of 763 new virus sequences belonging to the family *Mitoviridae* by metatranscriptomic analysis, the identification of previously uncharacterized mitovirus clades, and a putative new viral class. With this expanded understanding of the diversity of mitovirus and encoded RNA-dependent RNA polymerases (RdRps), we annotate mitovirus-specific protein motifs and identify hallmarks of mitochondrial translation, including mitochondrion-specific codons. This study expands the known diversity of mitochondrial viruses and provides additional evidence that they co-opt mitochondrial biology for their survival.

**IMPORTANCE:**

Metatranscriptomic studies have rapidly expanded the cadre of known RNA viruses, yet our understanding of how these viruses navigate the cytoplasmic milieu of their hosts to survive remains poorly characterized. In this study, we identify and assemble 763 new viral sequences belonging to the *Mitoviridae*, a family of (+)ssRNA viruses thought to interact with and remodel host mitochondria. We exploit this genetic diversity to identify new clades of *Mitoviridae*, annotate clade-specific sequence motifs that distinguish the mitoviral RdRp, and reveal patterns of RdRp codon usage consistent with translation on host cell mitoribosomes. These results serve as a foundation for understanding how mitoviruses co-opt mitochondrial biology for their proliferation.

## INTRODUCTION

RNA viruses are ubiquitous and prevalent components of the eukaryotic virosphere. However, the genetic diversity of eukaryotic RNA viruses is poorly described, due in part to sparse sampling and biases towards pathogens that impact human health or commercial agriculture. Metagenomics is a powerful approach to characterize viral biodiversity and has been used to substantially expand the known set of RNA viruses, particularly unculturable viruses from polar, marine, or microbiome contexts (1 - 5). However, there are still substantial gaps in our knowledge of viral biodiversity, particularly the holobionts of fungi and metazoans.

Positive single-stranded RNA viruses, or (+)ssRNA, in particular, exhibit a distinct ability to restructure the cytoplasm of host cells to facilitate their propagation. They remodel host endomembranes into invaginated viral replication organelles (ROs) derived from the endoplasmic reticulum, Golgi, or the outer mitochondrial or plastid membranes (6 - 10). Viral ROs concentrate viral RNA, proteins, and host factors to facilitate the formation of viral replication complexes (VRCs), as well as shield viral genome replication from host antiviral-sensing mechanisms (11, 12).

Beyond RO, some viruses have the ability to enter membrane-bound host organelles to directly access their biosynthetic potential, promote the formation of organelle-derived replication vesicles, or sequester organelle-localized proteins in the cytoplasm for their own benefit (9, 13, 14). Of the organelles linked to (+)ssRNA viral replication, mitochondria and chloroplasts maintain distinct chromosomes and gene expression machinery for the transcription, processing, and translation of organellar genes, including dedicated organellar ribosomes (15, 16). These features endow them with a unique capacity for nucleic acid metabolism and protein production (15 - 18). Mitochondria in particular are attractive targets for viral replication given their central roles in host defense mechanisms; the cloister of viral nucleic acid inside the mitochondrion itself may provide an opportunity to evade immune activation pathways at the outer mitochondrial membrane surface (19 - 21).

Metatranscriptomic sequencing studies are rapidly expanding the catalog of Earth’s RNA virome, yet our grasp of the strategies employed by viral proteins and RNA to hijack host organelles lags. Of particular interest are the viral families that have reported association with the mitochondria. Mitochondria and plastids are interesting targets for viral proliferation due to their dedicated gene expression pathway and incredibly dynamic membrane restructuring machinery. There have been recent advances giving insight into organelle replication and inheritance of nucleic acids, but few have investigated how these systems may be co-opted or perturbed by viral pathogens. We sought to gain a better understanding of the viral species that may interact with mitochondrial biology. As a first pass, we sought to exploit the unique endosymbiosis of mitochondria and their unique gene translation machinery.

We focused our study on the *Mitoviridae*, a family of (+)ssRNA viruses identified in association with fungal, plant, or invertebrate host mitochondria (17 - 21). Mitoviral genomes are composed of a capsidless single RNA with one open reading frame (ORF) encoding an RNA-dependent RNA polymerase (RdRp) (see Fig. 3A) (22). The presence of Mitovirus is linked to mitochondrial proteome remodeling in fungi and plants (23 - 25). While a handful of species (and many genomic fragments) have been identified in environmental samples, the mitoviral replicative cycle and the extent to which these viruses interact with host mitochondria in vivo remain poorly understood (23, 26, 27). The distribution and prevalence of *Mitoviridae* among Earth ecosystems are also unclear.

To determine the extent of mitoviral diversity and their evolutionary history, we searched metatranscriptomic data sets for evidence of mitochondria-associated (+)ssRNA viruses. In contrast to previous studies which focused on outer mitochondrial membrane remodeling by RNA viruses, we sought to identify viral genomes and/or genomic fragments that may co-opt the biosynthetic potential of mitochondrial matrix contents, such as the mitochondrial translational machinery, as a means of propagation.

## MATERIALS AND METHODS

### Searching SRA with Serratus

#### Lenarviricota protein query sequences

Mitovirus and other *Lenarviricota* nucleotide sequences were downloaded from GenBank with queries “txid186768[Organism:exp]” (*N* = 2,364, date: 12 October 2020) and “: txid2732407[Organism:exp] NOT txid186768[Organism:exp]” (*N* = 4,878, date: 12 October 2020), respectively, and hypothetical coding sequences were removed. To search short-nucleotide reads for mitoviral RdRP with a translated-nucleotide search using a standard genetic code, query CDS sequences were translated into amino acids using the standard genetic code to enable stop codon read-through (*transeq –Table 0*, EMBOSS 6.6.0).

#### Sequence Read Archive search space

Sequence Read Archive (SRA) sequencing runs were accessed from the SRA website using the search term: *‘"VIRAL METAGENOME" OR "VIROME" OR "VIROMIC" OR "VIRAL RNA" OR "METATRANSCRIPTOMIC" NOT "METAGENOMIC" NOT amplicon[All Fields] AND "platform illumina"[Properties] AND cluster_public[prop]’* on 25 October 2020, returning 60,327 runs, which were randomly sampled to 1,000 runs (Table S1).

#### Serratus search

Short-read sequencing runs were aligned against the above *Lenarviricota* protein query using the Serratus cloud-computing architecture (v0.2.0) (5) in protein mode. Reference architecture was 300 downloads (r5.xlarge) instances, 500 align (c5n.xlarge) instances, and 20 merges (c5.xlarge) instances. Translated-nucleotide search mode was run with DIAMOND (version 2.0.1) and parameters “*—unal 0 k 1 –b 0.2”*. Processing of all sequencing runs was attempted at least twice, and 981/1,000 (98.1%) were completed.

### Mitovirus discovery pipeline

#### Contig assembly

Selected SRA data sets from Serratus search were downloaded, unpacked, and all paired reads split using the SRA toolkit program fasterq-dump (Table S1). Sequencing reads were checked for sequencing barcodes using Trimmomatic sequencing adapter library (28) and trimmed accordingly. Contigs for each SRA experiment were then assembled using SPADES v.3.14.1 in RNA mode with default options (29, 30). For SRA experiment, ERR2195693 and ERR2809108 normal SPADES were used with default options due to both experiments containing unpaired reads.

#### Contig identification

The assembled contigs for each SRA experiment were then independently aligned with a reference set of viral RdRps derived from the NCBI protein database (see above) using the BLASTX functionality in Diamond v.2.0.6 (31), using the “--sensitive” tag, searching in all six frames, and requiring a minimum ORF of 300 amino acids. The contigs derived from each SRA project were searched using the vertebrate mitochondrial codon table (NCBI code 2), the fungal mitochondrial codon table (NCBI code 4), and the invertebrate mitochondrial codon table (NCBI code 5). Contigs that aligned against the mitovirus reference RdRps were pulled out using the AlignmentBreakup.py python file (https://github.com/TheLewisLab/Mitovirus-Code).

#### Mitovirus RdRp identification and confirmation

ORFs were identified using NCBI OrfFinder v.0.4.3 allowing for alternative start codons for codon tables 2 and 5due to the wide species diversity of mitochondrial start codons for those tables. Sequences with more than one ORF or ORFs less than 300 amino acids long were discarded. The resulting putative RdRps were then identified using BLASTP against the entire NCBI non-redundant protein database (accessed 23 February 2021). For sequences that aligned in multiple codon tables, fungal mitochondrial codon table 4 was used as mitoviruses are thought to mainly infect fungal hosts. The top BLASTP hit by E-value was then used to make the final taxonomy assignment for each putative viral sequence. While assembling the list of reference mitovirus sequences, we noticed a number of reference mitovirus sequences that had the highest percent identity to viruses other than mitoviruses and corrected them for the purpose of this study (Table S2).

### Read mapping and viral abundance

To estimate the abundance of each mitovirus in the metagenomic sample, sequencing reads were mapped back onto the assembled mitovirus contigs using Bowtie2 (32). A bowtie2 index was generated for all mitovirus genome segments found in each SRA sample, and bowtie2 v.2.4.5 was then used to map the original reads back onto the assembled genomes using default settings. The abundance of each mitovirus genome was calculated as mapped reads per kilobase per million total reads in the SRA experiment independent of any quality metrics or filtering. Estimates of viral abundance are reported in Table S2.

### RdRp clustering and phylogenetic analysis

RdRp amino acid sequences from this study and the RdRp amino acid sequences from NCBI-reported mitoviruses and their closest evolutionary neighbors, narnavirus, levivirus, and ourmiavirus, were compared by the Enzyme Function Initiative-Enzyme Similarity Tool (EFI-EST) (33, 34) to generate protein similarity networks with an E-value cutoff of 1 × 10^−5^ for class-level classification and of 1 × 10^−60^ for family-level classification consistent with previous studies (4, 35, 36). Reference sequences were downloaded from the NCBI protein database in May 2021 excluding partial protein sequences of fewer than 300 amino acids. RdRp clustering was represented using the Cytoscape organic layout (37). Sequences from the mitovirus cluster as well as from 10 randomly selected representative sequences from each clade noted above were then extracted and aligned using Clustal Omega multiple sequence alignment (38). The resulting alignment was then used to build a phylogenetic tree using FastTree v.2.1.11 with automatic determination of the substitution model and 1,000 ultrafast bootstraps and JTT+CAT model (39). The resulting tree was visualized using iTOL with clade branch lengths less than 0.9 collapsed, rooted at the most recent evolutionary neighbor between mitovirus sequences and the outgroups mentioned above (40).

### Codon usage analysis

Codon usage was calculated using the Python file codonfrequencyanalysis.py (GITHUB) using mitochondrial coding sequences in the NCBI reference sequence database using a mitochondrion and each organism filter tag. The prokaryotic viruses and their viral host coding sequences were obtained from the NCBI nucleotide database using corresponding organism tag and filtering for complete coding sequences. Nuclear codon usage was obtained using the HIVE Codon usage table search engine (41). Putative mitovirus codon usage correlation against the reference codon usage was calculated using Pearson’s R^2^ linear correlation formula as provided by the scipy python package. Heatmaps were created using the native R v.4.1.2 heatmap function with row hierarchical clustering based on the codon usage correlation values.

### Protein motif discovery

Protein motifs were identified using the de novo online motif discovery platform MEME with default parameters (42). The mitovirus-specific protein motifs were defined using the discriminative discovery mode in which the closest evolutionary neighbor viral RdRps from narnaviruses, ourmiaviruses, and leviviruses were designated as the outgroup (42). The percent occupancy at each motif location and the representative sequence alignment with evolutionary neighbors were visualized using clustal omega multiple protein alignment and Jalview (38, 43).

### Alphafold structural prediction

Alphafold mitovirus RdRp structural prediction of representative putative mitovirus RdRp sequence ERR3412979_288_4 was generated using the Google Collaboratory notebook distribution of AlphaFold and ColabFold with default parameters (44, 45). The mitovirus structural prediction and the location of protein motifs were visualized using PYMOL (46).

### RNA structural prediction

Putative mitoviral RNA structures were predicted using the RNAfold stand-alone binaries with minimum free-energy calculations on the last 100 nucleotides of the 3′ end of each mitovirus whose ORF contained a “stop” codon (47). Predicted RNA structures were then analyzed using MEME motif enrichment with a non-standard alphabet corresponding to the dot-bracket notation of the predicted RNA structures (42, 47).

## RESULTS

### Discovery of novel Mitovirus spp. and genomic fragments

**Fig 1:**
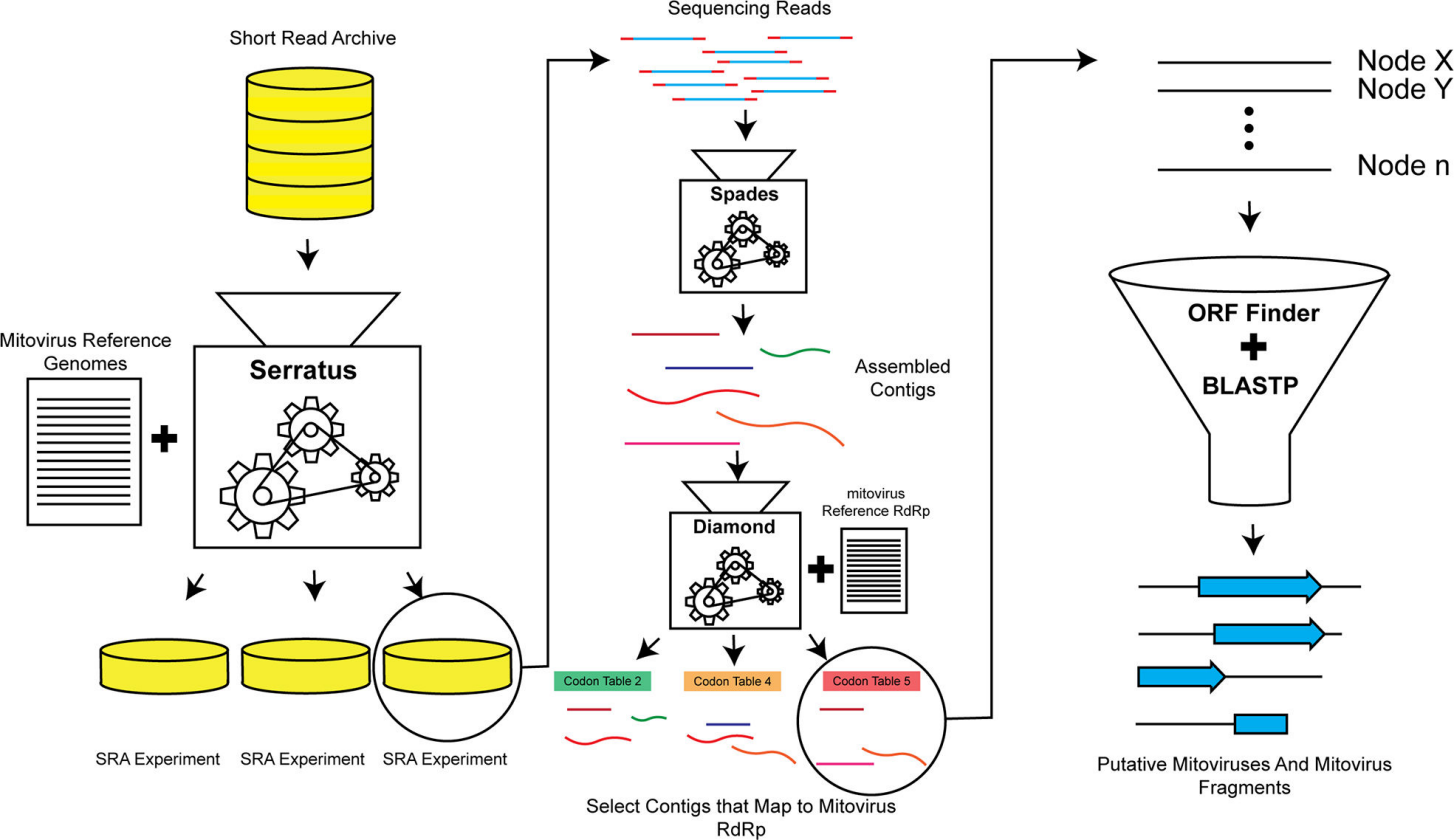
Schematic of computational pipeline used to discover putative new mitovirus sequences and sequence fragments.

**Fig 2:**
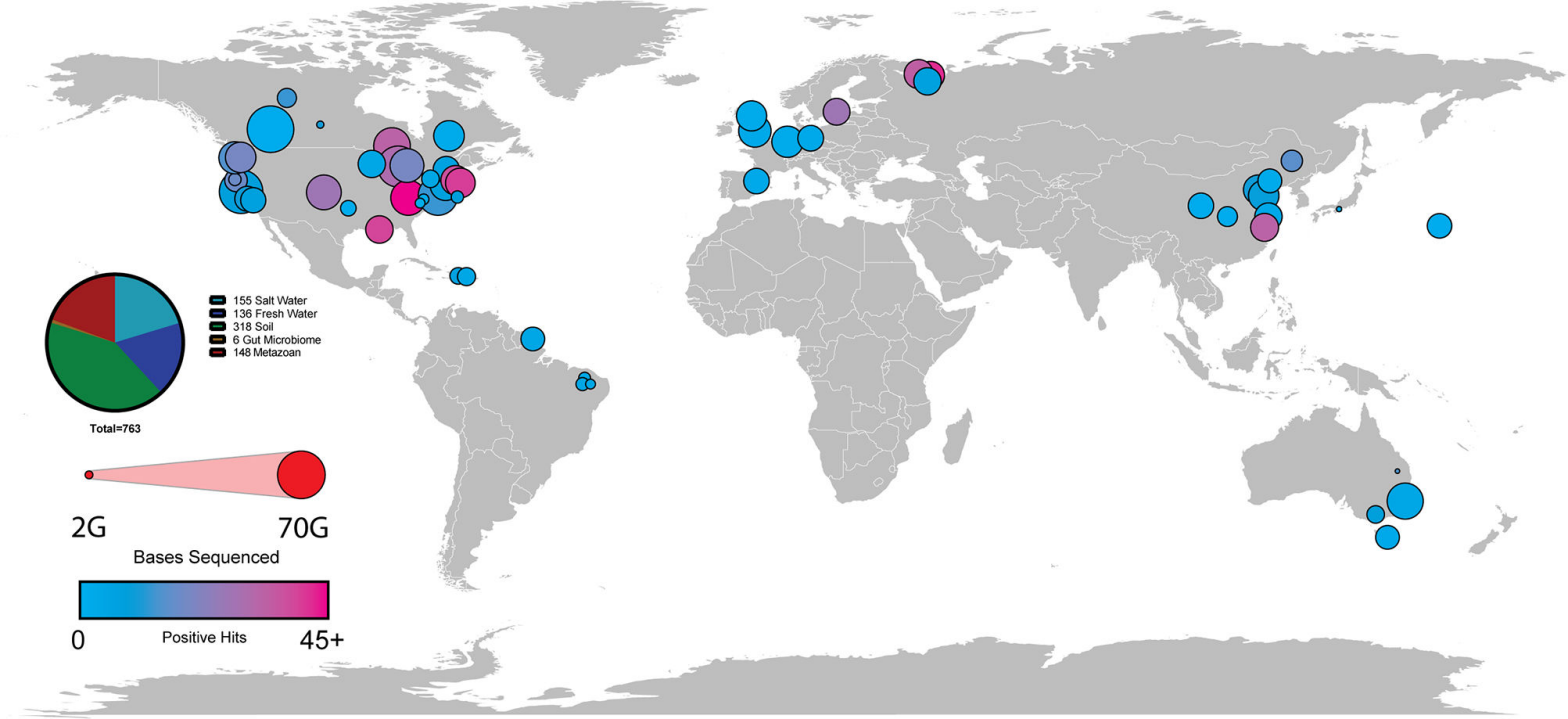
Diversity of sequencing sample collection sites. Map of all sample collection sites that resulted in the identification of new putative mitovirus. The size of each circle corresponds to the total number of bases sequenced at each site while the color reflects the number of new putative mitoviruses to come from that sample. A large amount of North American and Western European samples is a product of acquisition bias for samples in these regions.

**Fig 3:**
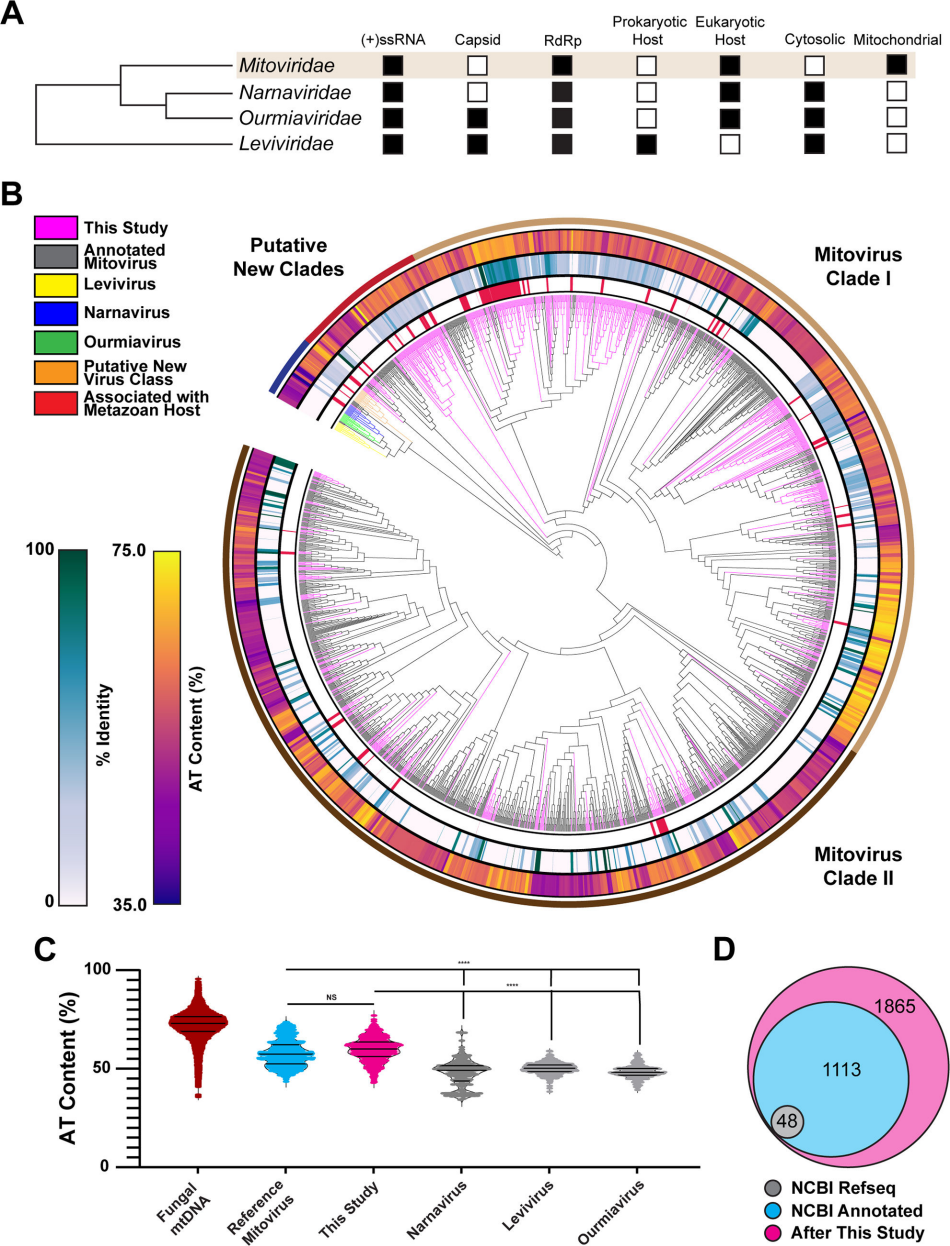
Discovery and characterization of novel putative mitovirus sequences. (A) Characteristics of *Mitoviridae* and its closest evolutionary neighbors: *Narnaviridae, Ourmiaviridae,* and *Leviviridae.* (B) Phylogenetic tree of new putative mitovirus sequences (magenta), existing mitovirus sequences as annotated by NCBI (black), a putative new viral clade (orange), and 10 representative sequences from each of the mitovirus’s closest evolutionary neighbors, the narnaviruses (blue), ourmiaviruses (green), and leviviruses (yellow). The AT content of each sequence, the percent identity to known mitovirus sequences, and whether the sequences were assembled from an animal sequencing project are represented by the concentric rings. (C) AT content of the new putative mitovirus sequences, reference mitoviruses, the mitovirus’s closest evolutionary neighbors, and fungal mitochondrial DNA. Statistical tests run: Mann–Whitney test, **** corresponds to *P*-value < 0.0001. (D) Number of mitovirus sequences before and after this study.

To gain a comprehensive understanding of the origins and evolution of mitoviruses, we used the Serratus viral discovery platform (5) to search sequencing reads from public metagenomic sequencing data sets hosted on the National Center for Biotechnology Information (NCBI) Sequence Read Archive (SRA) for mitoviral genomic sequences (see SRA Runs Searched; Table S1; Fig. 1) and identified 763 putative mitoviral genomes or genomic fragments of 900 base pairs or longer, sufficient to encode the mitoviral RdRp (Fig. 2). We used FastTree to perform phylogenetic analyses of these candidate mitoviruses, comparing them to previously reported mitoviral genomic fragments, using representative ourmiavirus, narnavirus, and levivirus sequences as outgroups (Fig. 3B). These evolutionary neighbors of *Mitoviridae* infect plants (ourmiaviruses), fungi (naraviruses), or bacteria (leviviruses) (22).

We scanned each assembled sequence contig for ORFs in all six translational contexts using the fungal, invertebrate, or vertebrate mitochondrial codon tables for ORFs that aligned with the amino acid sequence of a reference mitoviral RdRp using BLASTP. We assigned positive hits to taxonomy on the basis of BLASTP hits against the NCBI non-redundant protein database. Due to the non-traditional codon tables, existing viral discovery tools were not applicable.

While all candidates aligned with a previously reported mitoviral RdRp, the majority shared less than 45% sequence identity to a reference mitovirus (Table S2). A subset of divergent sequences was phylogenetically clustered; indeed, we uncovered two previously undescribed and phylogenetically distinct clades of sufficient divergence to constitute new viral families. Despite this genetic diversity, AT content among the mitoviral candidates was significantly higher than in ourmiaviruses, narnaviruses, or leviviruses, consistent with the reported reference mitoviral genomes and the mitochondrial genome content of potential eukaryotic hosts from these SRA projects (Fig. 3C). Overall this search expanded known mitovirus diversity by approximately 50% (Fig. 3D) (48, 49). Interestingly, mitoviral sequences associated with metazoan hosts cells tended to group in the phylogeny, potentially consistent with expansion into naive host niches. These findings imply that *Mitoviridae* are both prevalent and abundant within the global RNA virome and that substantial uncharacterized genetic diversity exists within this group.

### Evolutionary relationships among mitoviral clades

**Fig 4:**
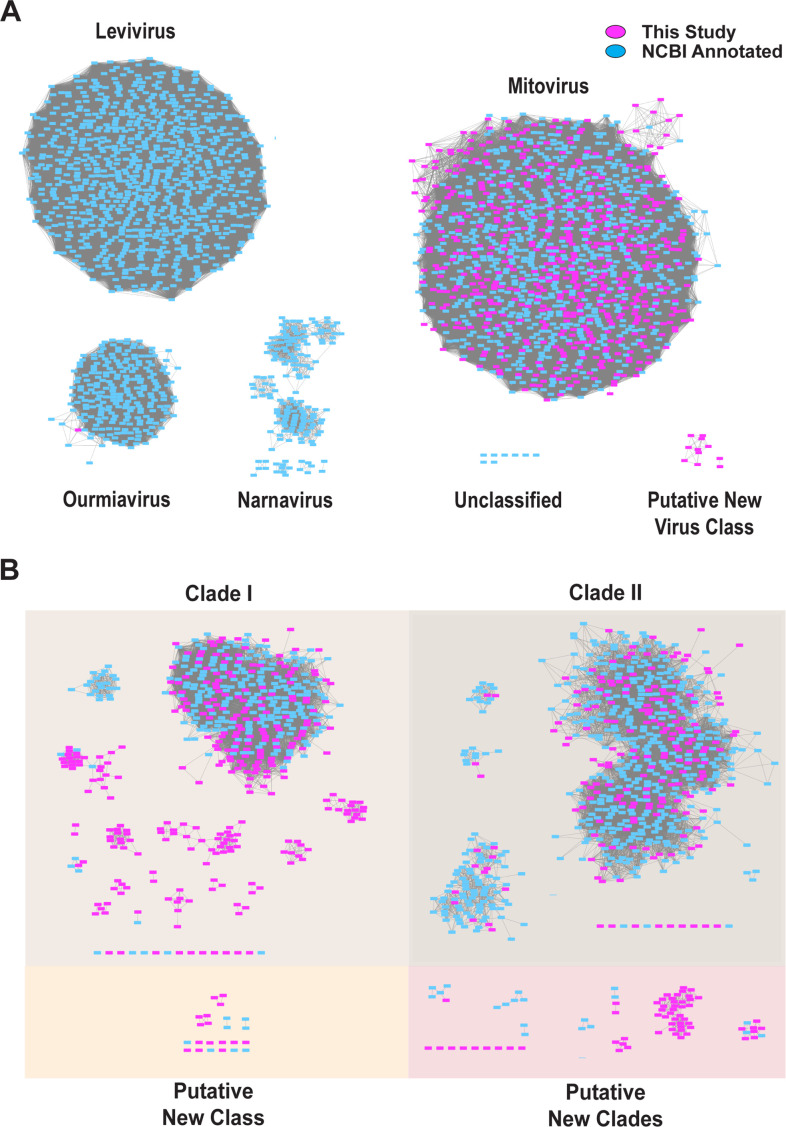
Sequence similarity networks of reference and putative new mitovirus RdRps. (A) Clade-level sequence similarity network of reference mitovirus and closest evolutionary neighbor RdRps (cyan), and putative new mitovirus RdRps (magenta) generated using EFI-EST with an E-value cutoff 1 × 10^−5^. (B) Family-level sequence similarity network of just mitovirus sequences generated using EFI-EST with an E-value cutoff of 1 × 10^−60^.

We next examined the potential sequence and function space of the mitoviral RdRp sequences we identified using sequence similarity networks (SSNs). SSNs are useful tools to study the relationships between large sets of protein sequences that may be hard to root in traditional phylogenetic trees when there exist large gaps in characterized genetic diversity among sampled sequences. For SSN analysis, we extracted all RdRps from the phylum *Lenarviricota* which is made up of the family *Mitoviridae* and their closest evolutionary neighbors, *Narnaviridae*, *Leviviridae*, and *Ourmiaviridae* from the NCBI protein database, and aligned them pairwise using EFI-EST (33). The resulting alignments were then used to build SSNs using EFI-EST, implemented, and visualized in Cytoscape (33, 37). Furthermore, we noted a distinct cluster formed exclusively by sequences without clear homologs in the NCBI-nr database, suggesting a novel phylogenetic group (Fig. 4A).

We next compared these results to an SSN constructed using an E-value cutoff of 1 × 10^−60^, which has been used to split viruses into family-level assignments (4, 35, 36) (Fig. 4B). We identified 17 as yet uncharacterized family-level clusters. This analysis also suggested that the family *Mitoviridae* may consist of two major clades that are well represented in known mitovirus diversity (Fig. 3B and Fig. 4B). The results from the phylogenetic classification and SSN analysis suggest there exist a large number of previously unidentified family-level clusters that make up the family *Mitoviridae,* many of which seem to be associated with metazoan hosts.

### Discovery of conserved structural motifs in the mitoviral RdRp

**Fig 5:**
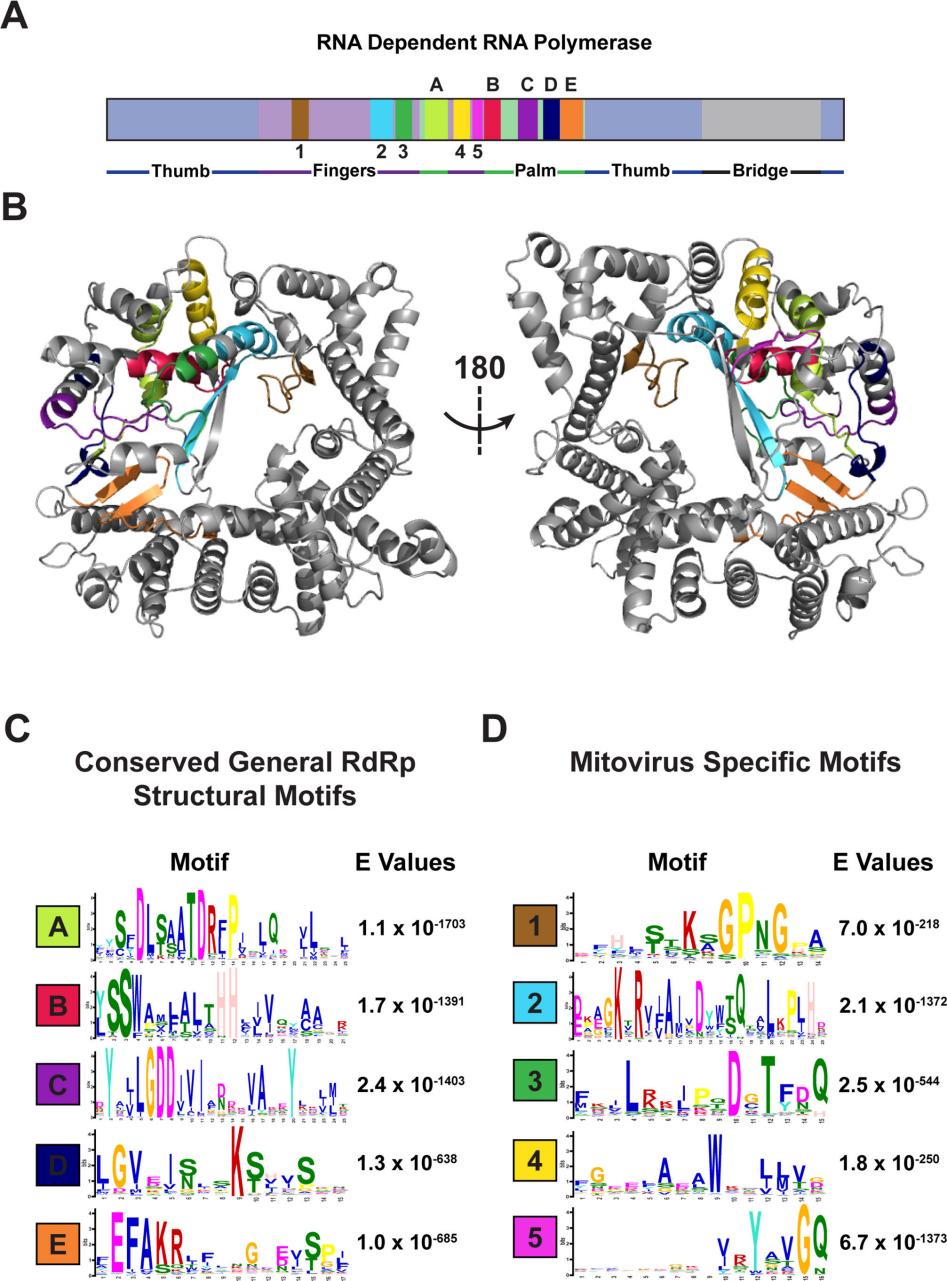
Mitovirus-conserved protein motifs. (**A**) Location of each identified protein motif relative to known RNA-dependent RNA polymerase domains. (**B**) Alphafold structural prediction and corresponding motif location on representative mitovirus RdRp. (**C**) Conserved general RdRp catalytic motifs discovered using standard MEME motif discovery platform (42). (**D**) Conserved mitovirus unique protein motifs as reported by MEME discriminatory mode using narnavirus RdRps as outgroup.

Viral RdRps can generally be identified by five evolutionarily conserved structural motifs located in the core of the RdRp (34, 38). These protein motifs play a central role in catalysis and therefore retain high structural similarity across all five Baltimore groups of viral RdRps (34, 38). To characterize the conserved catalytic domains within mitoviral RdRps, as well as interrogate unique structural motifs, we used the sequence motif discovery platform MEME (50). We searched all 763 newly identified mitoviral peptide sequences for conserved domains and successfully identified the five conserved general RdRp catalytic motifs (Fig. 5C). These motifs retained all catalytically required amino acids such as the characteristic DX2-5D and GDD motifs and were spatially organized consistent with previously identified viral RdRp domains (Fig. 5A and C).

Using the Google Research Colaboratory distribution of AlphaFold and ColabFold (51, 52), we were then able to get a structural prediction of a representative mitovirus RdRp (Fig. 5B; Fig. S1). By mapping the conserved general RdRp catalytic motifs onto the structural prediction, we observed that all motifs fall within the catalytic pocket of the RdRp, with the highly conserved acids pointing inward (Fig. S1B), suggesting that mitoviral RdRps are indeed catalytically active.

Next, we sought to identify structural motifs unique to the *Mitoviridae* family. To do so, we used the discriminatory mode of MEME to search for conserved protein motifs enriched in the mitoviral RdRp sequence set relative to their closest evolutionary neighbor, the *Narnaviridae* (19). We uncovered five unique highly conserved structural motifs near the core of the RdRp (Fig. 5A and D). To confirm that these sequence motifs are unique to the *Mitoviridae* family, we performed simple enrichment analysis in the MEME suite (50), in which the mitovirus evolutionary neighbors, narnaviruses, leviviruses, and ourmaiviruses, are searched for occurrences of the mitovirus-specific motifs. This analysis revealed no significant matches, indicating these protein motifs are truly unique to mitovirus RdRps. A representative multiple sequence alignment between these four groups is shown in Fig. S2. Mapping these motifs onto the ColabFold structural prediction revealed they are located in the core of the RdRp (Fig. 5B; Fig. S1). Mitovirus-specific motifs 1, 2, and 3 are all facing the interior of the RdRp core with highly conserved amino acids indicating a possible role in catalysis (Fig. S1C). In contrast, motifs 4 and 5 are solvent exposed on the exterior of the RdRp, consistent with a possible role in cofactor recruitment (Fig. S1D and E).

### Evidence for mitoribosomal translation of the mitoviral RNA-dependent RNA polymerase

**Fig 6:**
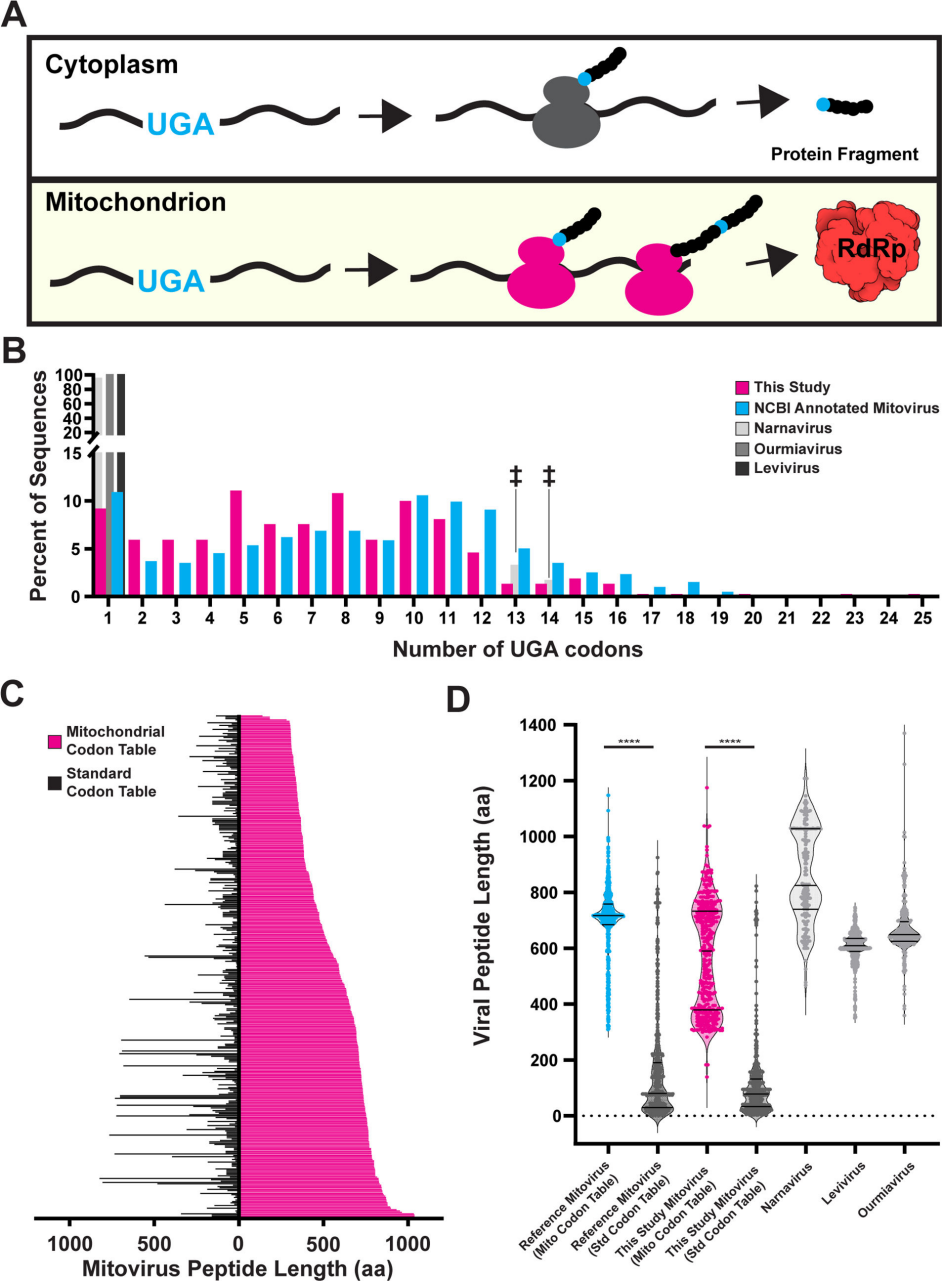
Analysis of mitovirus non-canonical codon usage. (**A**) Transcripts using mitochondrial-specific UGA codon will only produce full-length products if translated on mitochondrial ribosomes. (**B**) Number of UGA codons in putative new mitovirus sequences (magenta), reference mitovirus sequences (cyan), and closest evolutionary neighbors (grays). ‡ represent misannotated narnavirus sequences (see Methods and Table S2). (**C**) Length of putative mitovirus peptides if translated using either the mitochondrial codon table (magenta) or standard cytosolic codon table (black). (**D**) Violin plot of data in B, including reference mitoviruses (cyan), and closest evolutionary neighbors (grays). Statistical tests run: Mann–Whitney test, **** corresponds to *P*-value < 0.0001.

Mitoribosomes function in the mitochondrial matrix and employ a codon table distinct from their nuclear counterpart (15). These non-canonical codons are decoded using tRNAs encoded in the mitochondrial genome (15, 53). While the exact encoding of amino acids varies from species to species, one hallmark of mitochondrial translation is the decoding of the UGA trinucleotide as tryptophan instead of a “stop” codon (15, 53). This distinction can be leveraged to identify proteins that are truncated when translated on cytoplasmic ribosomes at full length when translated in the mitochondrial compartment (Fig. 6A). We wondered to what extent mitoviruses use the mitochondrial-specific codon and its effect on the mitovirus RdRp. We found that roughly 55% of previously reported mitovirus and 48% of our new putative mitovirus sequences decoded UGA as tryptophan instead of “stop” with the majority encoding this tRNA multiple times (Table S3; Fig. 6B). We estimate that this decoding is required for the expression of the full-length RdRp (Fig. 6C and D).

The encoding of amino acids between all synonymous triplicate nucleotide codons is not equal, and this codon usage bias (CUB) can often be useful in tracking evolutionary history, differential gene expression, and even virus–host interactions (50, 54 - 58), making CUB potentially useful to restrict the breadth of potential hosts (51, 54, 57) (Fig. S3). We probed whether codon usage among mitoviral sequences was more similar to that of mitochondrially encoded or nuclear-encoded gene products. To test this, we calculated the Pearson’s correlation coefficients between the codon usage frequency of each putative mitoviral RdRp or mitoviral RdRp fragment and a range of different host mitochondrial and nuclear codon usage frequencies as defined in Table S4.

**Fig 7:**
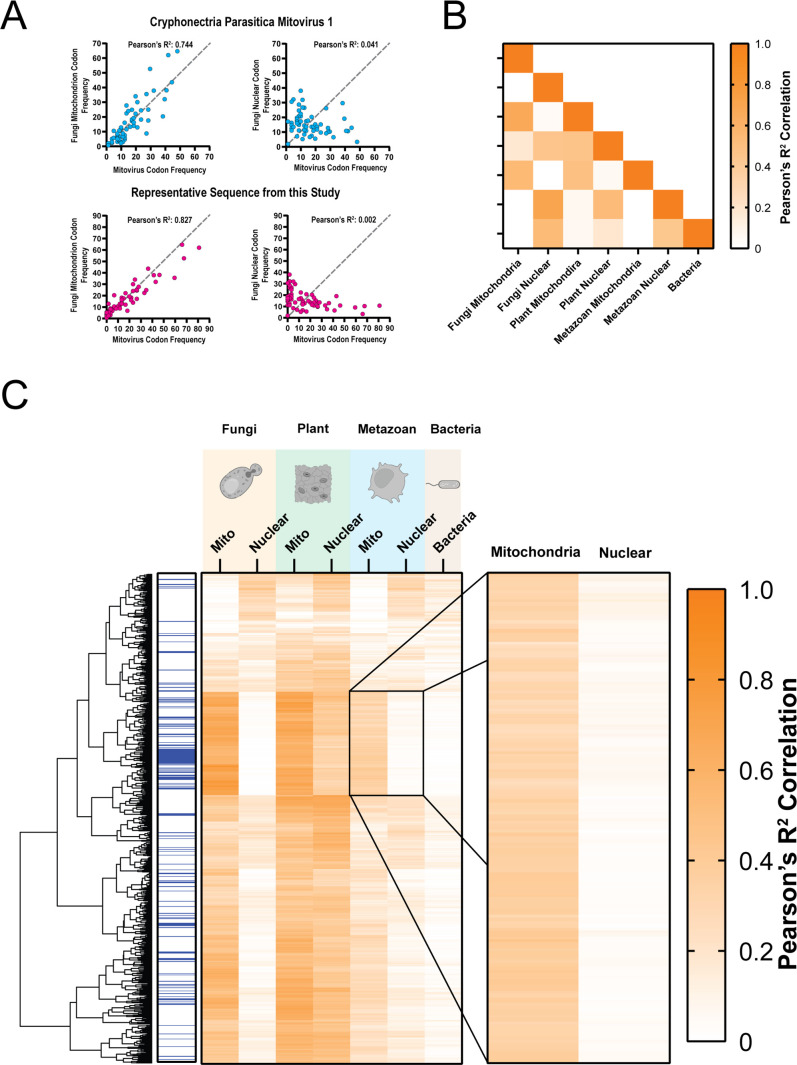
Codon usage bias of mitovirus sequences. (**A**) Codon usage correlation of representative putative new mitovirus (magenta) and reference mitovirus (cyan) between fungal nuclear codon usage and fungal mitochondrial codon usage. (**B**) Heatmap of codon usage correlation values between every reference data set used. (**C**) Heatmap of codon usage correlation values for each putative new mitovirus open reading frame (row) and both the mitochondrial and nuclear codon usage for fungal, plant, metazoan, and bacteria (columns). Sequences associated with animal sequencing studies are highlighted in blue. A subset of sequences with high metazoan codon usage correlation was called out. All correlation values are Pearson’s linear R^2^.

We found that the codon usage frequencies of putative novel mitoviral RdRps and RdRp fragments correlate significantly more (*P* < 0.0001) with that of the fungal mitochondria than the nuclear transcripts of its hosts (Fig. 7A and C; Fig. S4A). The majority of identified mitoviruses have been identified in association with fungal hosts. Of the previously identified 46 reference mitovirus sequences, 45.6% were discovered in the fungal Ascomycota phylum, 19.6% in the Basidiomycota phylum, 17.4% in Mucoromycota phylum, 4.3% in plants, and 13.0% assembled from non-specific metagenomic data. Consistently, the majority of the mitovirus sequences that we report here display a pattern of codon usage most similar to fungi and specifically with the fungal mitochondrial codon usage table. We also here report a set of mitovirus sequences that when translated display a high codon usage correlation with the invertebrate mitochondrial table.

In contrast, codon usage among narnaviral RdRps, which exclusively replicate within the cytoplasm of the host cell, was significantly more correlated (*P* < 0.0001) with nuclear codon usage than it was with mitochondrial codon usage (Fig. S3A or Fig. S4A). Interestingly, codon usage in the mitoviral RdRps also showed a strong correlation with both the plant mitochondrial and nuclear codon frequencies (Fig. 7C; Fig. S4A). This could suggest that mitovirus RdRps are poised to replicate both in the mitochondria and cytoplasm of the plant host. However, plant mitochondrial codon usage is extremely similar to both fungal mitochondrial codon usage and plant nuclear codon usage (Fig. 7B; Fig. S4C), suggesting that this high codon usage correlation could also be indicative of this close association. We also identified a subset of RdRps that showed a significant correlation with the metazoan mitochondrial codon usage table (*P* < 0.0001) (Fig. 7C; Fig. S4B). Interestingly, this subset is also enriched for mitovirus sequences assembled from animal sequencing projects (Fig. 7C in blue). This supports recent reports identifying mitoviruses in invertebrate metagenomic samples (52). Taken together, these findings strongly suggest that mitovirus RdRps utilize the mitochondrial ribosomes and their unique subset of tRNAs for mitoviral translation.

## DISCUSSION

Inspired by the recent success in searching metagenomic sequencing data sets for novel virus species (1 - 59 - 60 - 60), here we identified 763 new putative mitovirus sequences and sequence fragments from publicly available metagenomic profiles of samples isolated from a wide array of geographic locales and ecological environments (Fig. 2). This study increases the number of known mitovirus sequences, with an approximate 50% increase in diversity. Our findings underscore the scarcity of knowledge about eukaryotic ssRNA viruses and the understudied *Mitoviridae* family in particular. Furthermore, this study serves as an initial foray into exploring how mitoviruses may exploit the unique organelle biology of host cells for their propagation.

Here, we expand the understanding of the evolutionary relationships among ssRNA viruses and the relationships between new and previously identified mitoviruses. A number of our newly identified mitoviruses cluster into distinct family-level organizations, suggesting a broader genetic diversity amongst the clade than had been appreciated in previous studies (22). Indeed, SSN and phylogenetic analyses indicated that the family *Mitoviridae* actually consists of two distinct major clades, with evidence suggesting many other underrepresented family-level clusters (Fig. 3B and Fig. 4B).

Previous reports on mitoviruses tend to rely primarily on crude mitochondrial fractionations as evidence for their mitochondrial localization and do not show a functional relationship between the mitovirus and the mitochondrial gene expression systems (26, 27). It has also been shown mitoviruses are able to horizontally transfer between fungal species by presumable mitochondrial fusion during protoplast fusion events (61, 62). However, there still lacks a direct link between the mitovirus life cycle and mitochondrial biology. Here, through codon usage correlations and mitochondrial codon analysis, we provide evidence linking mitoviruses to the mitochondrial gene expression systems. Our data suggest that not only do the majority of described mitoviruses rely on mitochondrial ribosomes for RdRp translation but mitoviral codon usage parallels that of the host cell, suggesting an evolutionary adaptation to hijack the mitochondrial gene expression system (Fig. 6 and 7).

It is well documented that (+)ssRNA virus remodel endogenous host membranes to form ROs and recruit necessary host factors that make up their VRC (6, 7, 9 - 11). Interfacing with host factors requires functional structural motifs within the viral RdRp to facilitate membrane remodeling and protein recruitment (63). The field currently lacks robust cytological data as to the subcellular localization of mitoviruses within intact host cells, and therefore knowledge about how they accomplish this process is limited. Given their unique association with mitochondria, we wondered if *Mitoviridae* may utilize specific protein motifs or structures that make them distinct from all other previously characterized RNA viruses that replicate in the cytoplasm. Here, we identify five previously undescribed evolutionary conserved protein motifs unique to the polymerases of family *Mitoviridae*, likely implicated in that process (Fig. 5). Structural predictions of a typical mitovirus RdRp suggest that two of these identified protein motifs map to the surface of the folded protein, rendering them accessible to host cofactors or protein recognition domains (Fig. S1D and E). Beyond their potential role in catalyst or host factor recruitment, these novel mitovirus-specific motifs will also serve as useful for future studies looking to identify new mitoviruses. While outside the scope of this study, future experiments addressing the function of these conserved mitovirus-specific domains would be of great interest.

Beyond their interest as a virus that may exploit mitochondrial-specific gene expression equipment, mitoviruses also represent a potentially exciting development for efforts towards mitochondrial transgenesis. A notable barrier to extant methods for genome engineering in the mitochondria has been the lack of tools to introduce endogenous nucleic acid into the mitochondrial matrix (64, 65). Just as the discovery of plasmids allowed for exogenous gene expression, and the characterization of the yeast 2-μm plasmid supported the expansion of yeast genetic editing, a better understanding of mitovirus biology may yield fruitful insights for manipulating organellar nucleic acids.

Through searching publicly available sequencing data, we have expanded the known mitovirus diversity and shed some light on their interactions with mitochondria. While there is much to still uncover surrounding the molecular and cell biology of mitoviruses, this study serves as the first foray into an understudied world.

## SUPPLEMENTAL MATERIAL

Table S1 msystems.01002-22-s0001.xlsx SRA projects analyzed in this study with data citation information

Table S2 msystems.01002-22-s0002.xlsx Assembled mitovirus sequences and associated metadata

Table S3 msystems.01002-22-s0003.xlsx Codon usage analysis reference sets

Table S4 msystems.01002-22-s0004.xlsx NCBI IDs of fungal genomes searched for integrated mitovirus sequences

FIG S1 msystems.01002-22-s0005.tif Alphafold Prediction of representative mitovirus RdRp. (A) Location of each subpanel and conserved protein motif (Fig. 2) on representative mitovirus RdRp structural prediction. (B) Closer look at predicted catalytic pocket of mitoviral RdRp with highly conserved/catalytically essential amino acids shown. (C) Closer look at mitovirus specific protein motifs located within predicted catalytic pocket. (D/E) Closer look at mitovirus specific protein motifs predicted to be located on the surface of the RdRp structure. Structural predictions done on putative mitovirus ERR3412979_288_4 using Colabfold and visualized in PyMOL.

FIG S2 msystems.01002-22-s0006.tif Representative multiple sequence alignment of mitoviral RdRp and closest evolutionary neighbors. Multiple sequence alignment using Clustal Omega was performed on three mitovirus RdRps and representative RdRps from mitoviral closest evolutionary neighbors the ourmiaviruses, leviviruses, and narnaviruses. Motifs correspond to motifs in Fig. 4, Black amino acids are highly conserved amino acids from motif analysis.

FIG S3 msystems.01002-22-s0007.tif Narnavirus and protist virus codon usage correlations. (A) Codon usage correlation between representative narnavirus and fungal mitochondrial codon usage (left) and fungal nuclear codon usage (right). (B) Example codon usage correlation between four protist viruses and their respective host codon usage. All correlation values are Pearson’s linear R^2^.

Fig S4 msystems.01002-22-s0008.tif Extended codon usage analysis. (A) Violin plot of codon usage correlation values from Fig. 4C for reference mitoviruses (cyan), new putative mitoviruses (magenta), and narnaviruses (black). (B) Violin plot of codon usage correlation values of called out mitoviruses in Fig. 4C. (C) Codon usage correlation between plant mitochondrial codon usage and plant nuclear codon usage. Statistical tests run: (A) Two-Way ANOVA with Tukey’s multiple comparison post hoc, (B) Mann-Whitney, **** corresponds to *P* value < 0.0001.

ASM does not own the copyrights to Supplemental Material that may be linked to, or accessed through, an article. The authors have granted ASM a non-exclusive, world-wide license to publish the Supplemental Material files. Please contact the corresponding author directly for reuse.
