# Supplementary figures and images for: Metatranscriptomic analysis uncovers prevalent viral ORFs compatible with mitochondrial translation
Source: mSystems. 2023 May 18;8(3):e01002-22. doi: 10.1128/msystems.01002-22 (PMC10308907; doi:10.1128/msystems.01002-22)

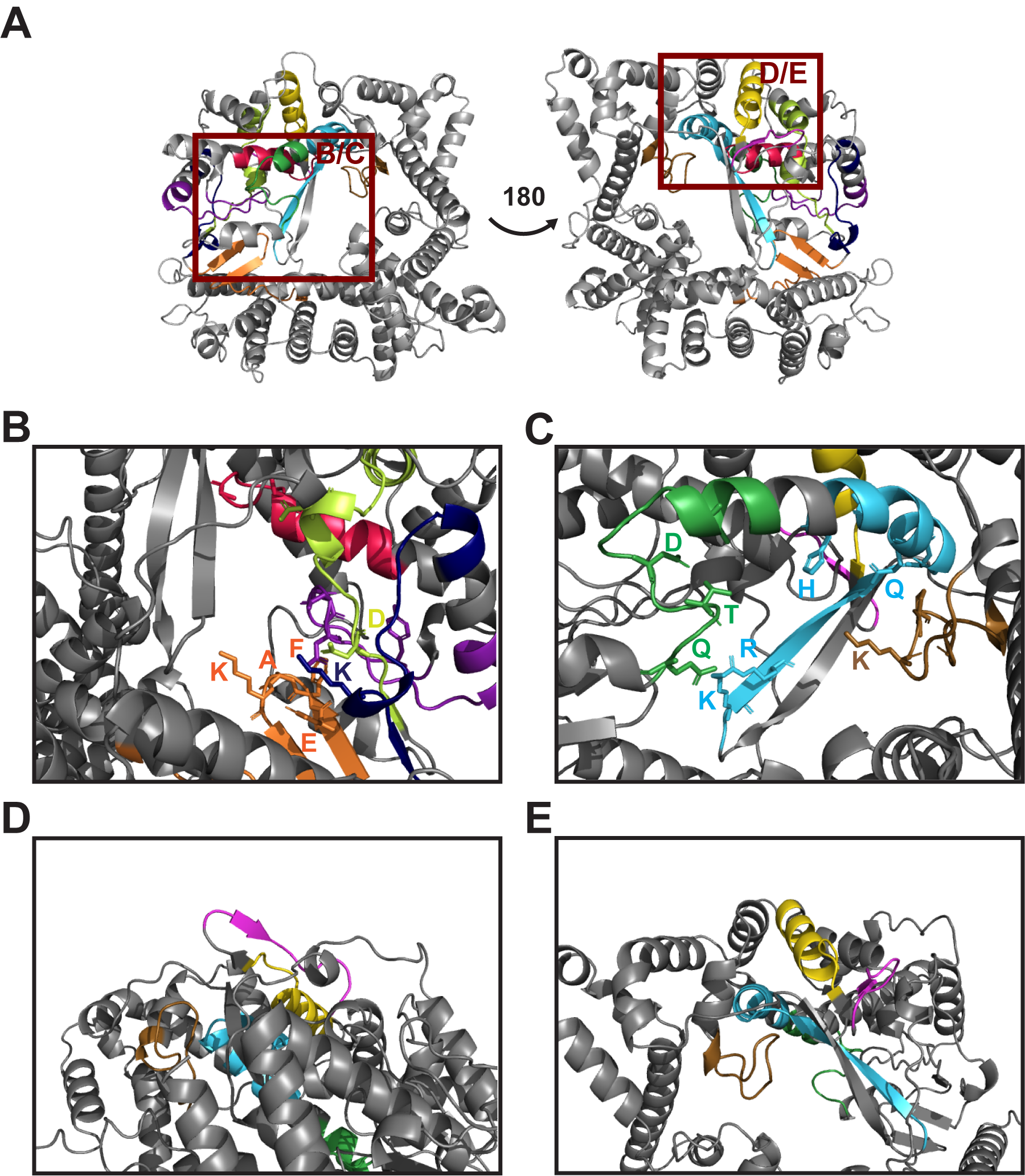

Supplement: FIG S1 — Alphafold Prediction of representative mitovirus RdRp. (A) Location of each subpanel and conserved protein motif (Fig. 2) on representative mitovirus RdRp structural prediction. (B) Closer look at predicted catalytic pocket of mitoviral RdRp with highly conserved/catalytically essential amino acids shown. (C) Closer look at mitovirus specific protein motifs located within predicted catalytic pocket. (D/E) Closer look at mitovirus specific protein motifs predicted to be located on the surface of the RdRp structure. Structural predictions done on putative mitovirus ERR3412979_288_4 using Colabfold and visualized in PyMOL. [file msystems.01002-22-s0005.tif]

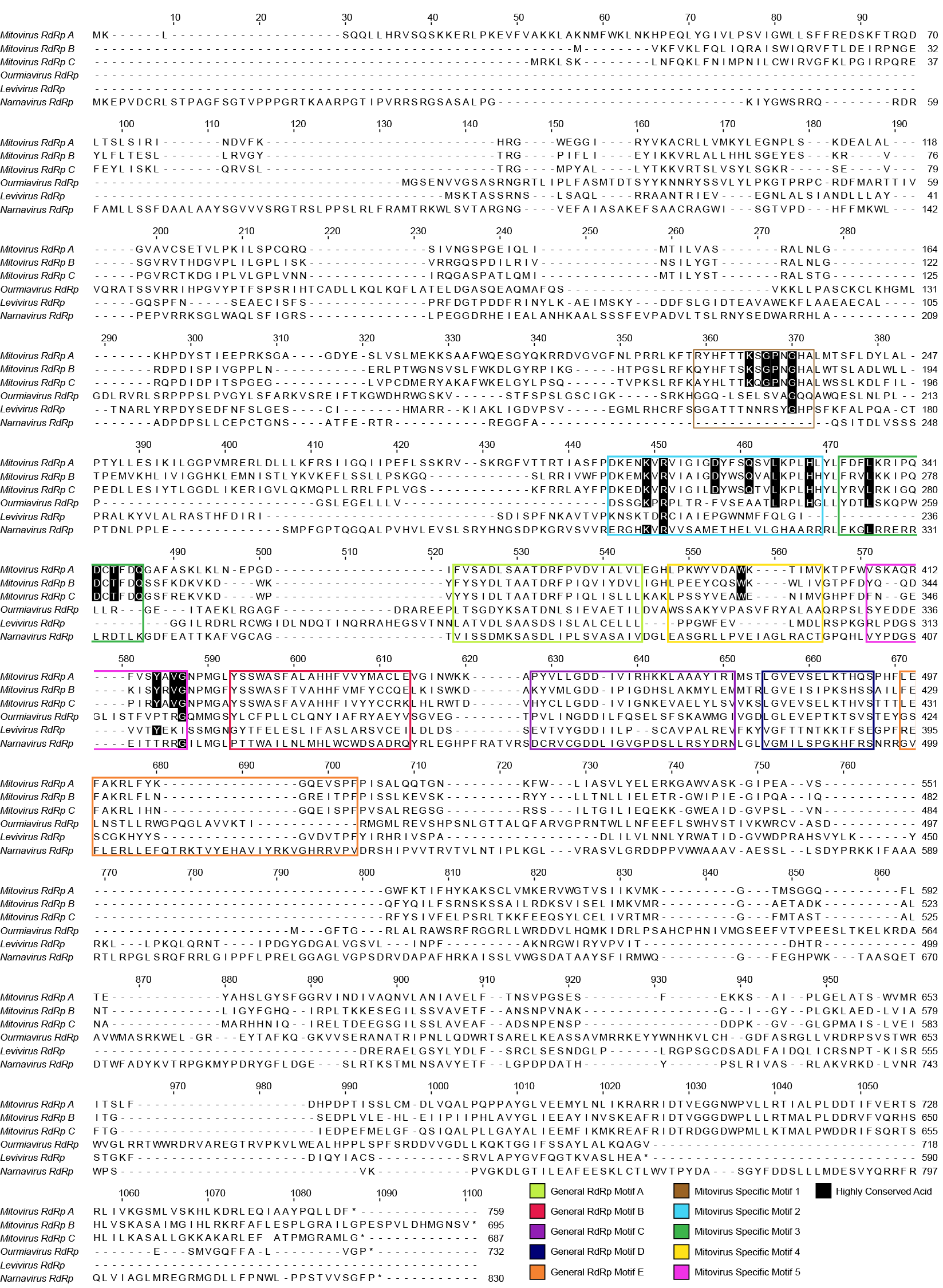

Supplement: FIG S2 — Representative multiple sequence alignment of mitoviral RdRp and closest evolutionary neighbors. Multiple sequence alignment using Clustal Omega was performed on three mitovirus RdRps and representative RdRps from mitoviral closest evolutionary neighbors the ourmiaviruses, leviviruses, and narnaviruses. Motifs correspond to motifs in Fig. 4, Black amino acids are highly conserved amino acids from motif analysis. [file msystems.01002-22-s0006.tif]

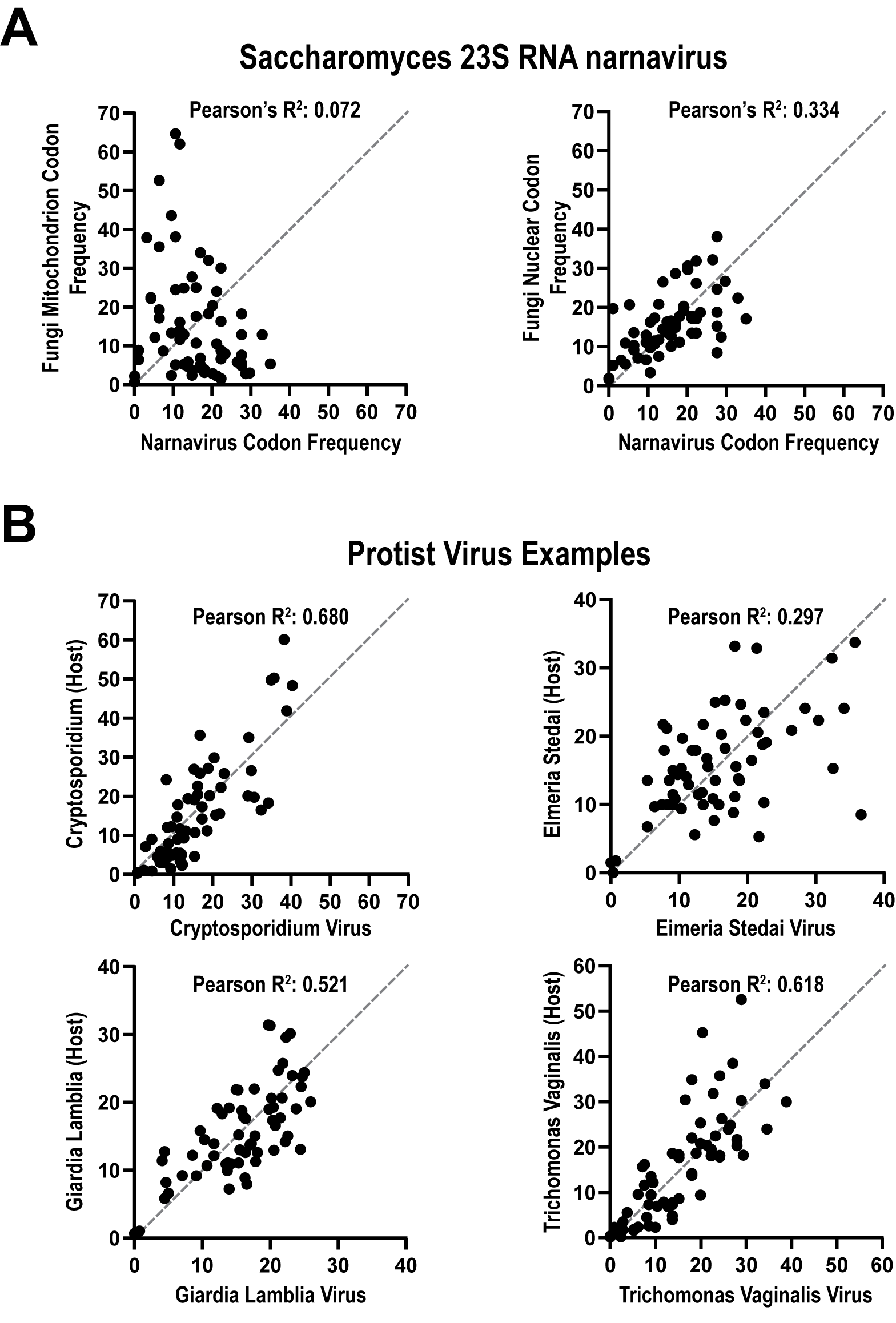

Supplement: FIG S3 — Narnavirus and protist virus codon usage correlations. (A) Codon usage correlation between representative narnavirus and fungal mitochondrial codon usage (left) and fungal nuclear codon usage (right). (B) Example codon usage correlation between four protist viruses and their respective host codon usage. All correlation values are Pearson’s linear R2. [file msystems.01002-22-s0007.tif]

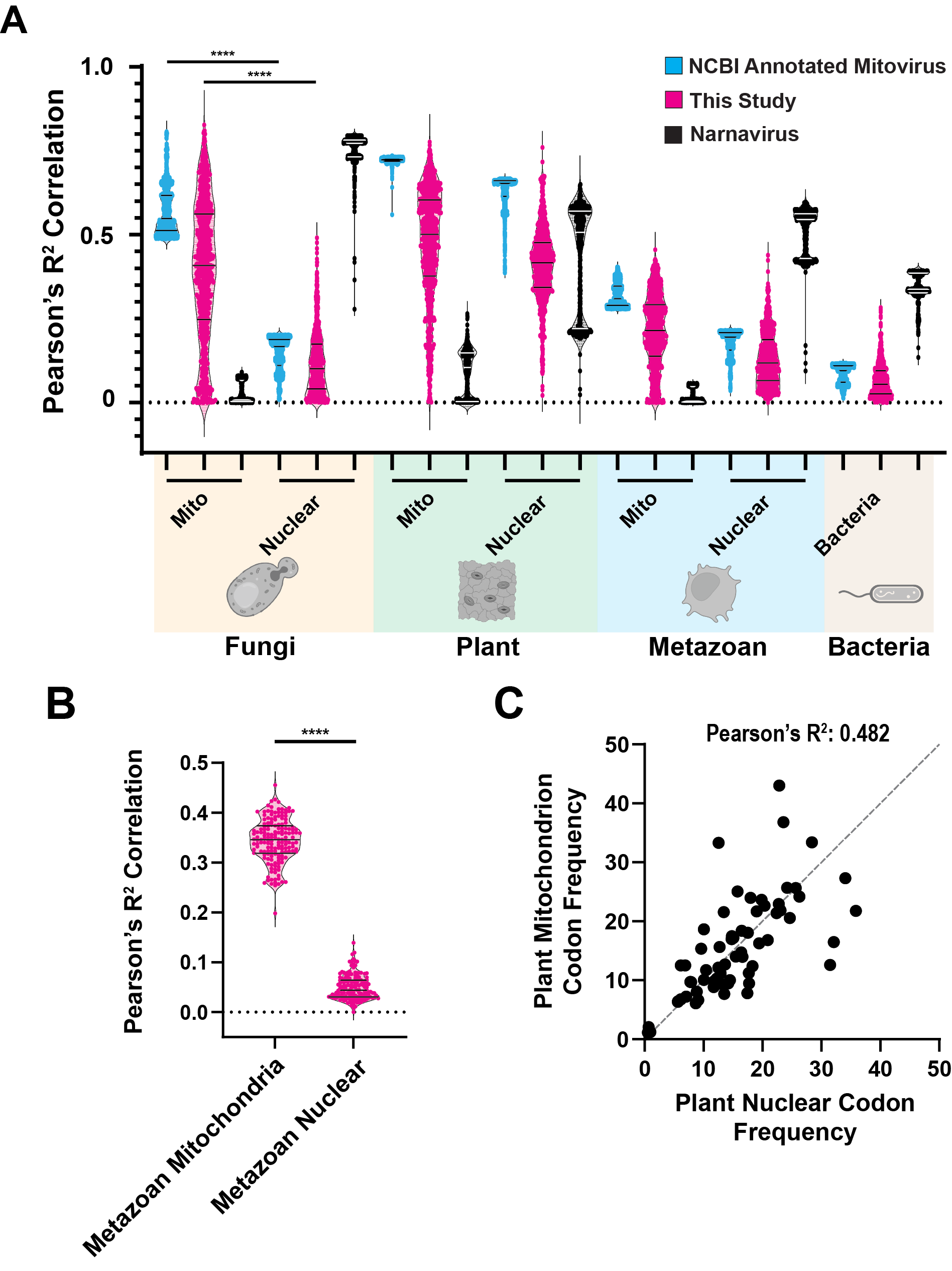

Supplement: Fig S4 — Extended codon usage analysis. (A) Violin plot of codon usage correlation values from Fig. 4C for reference mitoviruses (cyan), new putative mitoviruses (magenta), and narnaviruses (black). (B) Violin plot of codon usage correlation values of called out mitoviruses in Fig. 4C. (C) Codon usage correlation between plant mitochondrial codon usage and plant nuclear codon usage. Statistical tests run: (A) Two-Way ANOVA with Tukey’s multiple comparison post hoc, (B) Mann-Whitney, **** corresponds to P value < 0.0001. [file msystems.01002-22-s0008.tif]
